# Initiation of human mammary cell tumorigenesis by mutant KRAS requires YAP inactivation

**DOI:** 10.1038/s41388-019-1111-0

**Published:** 2019-11-26

**Authors:** Sylvain Lefort, Susanna Tan, Sneha Balani, Bo Rafn, Davide Pellacani, Martin Hirst, Poul H. Sorensen, Connie J. Eaves

**Affiliations:** 1 Terry Fox Laboratory, British Columbia Cancer Agency, 675 West 10th Avenue, Vancouver, BC V5Z 1L3 Canada; 2 Department of Molecular Oncology, British Columbia Cancer Agency, 675 West 10th Avenue, Vancouver, BC V5Z 1L3 Canada; 3 Canada’s Michael Smith Genome Sciences Centre, British Columbia Cancer Agency, Vancouver, BC V5Z 1L3 Canada; 4 Department of Microbiology and Immunology, Michael Smith Laboratories, University of British Columbia, Vancouver, BC V6T 1Z4 Canada

**Keywords:** Breast cancer, Cancer models, Cancer stem cells

## Abstract

High YAP activity is associated with poor prognosis human breast cancers, but its role during the initial stage of mammary cell transformation is unknown. To address this question, we designed experiments that exploit the ability of *KRAS*^*G12D*^-transduced subsets of freshly isolated normal human mammary cells to form invasive tumors rapidly and efficiently when transplanted into immunodeficient mice. Initial examination of the newly developing tumors thus generated revealed a consistent marked loss of nuclear YAP, independent of the initial primary human mammary cell type transduced. Conversely, co-transduction of the same subsets of primary human mammary cells with *KRAS*^*G12D*^ plus the constitutively active *YAP*^*S127A*^ prevented tumor formation. These findings contrast with the enhanced display of transformed properties obtained when the immortalized, but non-tumorigenic MCF10A cells are transduced just with *YAP*^*S127A*^. In addition, we show that *YAP*^*S127A*^-transduction of the human MDA-MB-231 breast cancer cell line (that carry a similar *KRAS* mutation) enhances their metastatic activity in vivo. We also discover that the *KRAS*^*G12D*^*-*induced early loss of YAP in primary human mammary cells is associated with their induced secretion of amphiregulin. Collectively, these findings suggest that YAP can differentially affect the acquisition of malignant properties by human mammary cells at different stages of their transformation.

## Introduction

Yes-associated protein (YAP) and tafazzin (TAZ) are sometimes found in the cytoplasm as well as in the nucleus of many cell types where they act in concert with TEAD co-factors to form complexes that regulate gene transcription [1, 2]. The control of nuclear accumulation of YAP/TAZ is thus a key determinant of their function as transcriptional regulators. YAP and TAZ are downstream effectors of the Hippo pathway, and the phosphorylation of YAP by the Hippo-activated large tumor suppressor (LATS) kinase leads to the sequestration of YAP in the cytoplasm and hence suppression of its transcription factor activity [2–4]. YAP/TAZ activity is also modulated by cellular interactions with the extracellular matrix that induce the formation of F-actin stress fibers and a consequent stimulation of YAP/TAZ transfer to the nucleus. Conversely, when cell adhesion is restricted, nuclear levels of YAP/TAZ are reduced [5, 6].

Numerous studies have shown that YAP and TAZ can variably regulate the proliferation, progression, migration, and metastasis of malignant human cells [7]. Specifically relevant to the present study is the finding of high levels of YAP expression in invasive lobular breast cancer, and an association of increased nuclear localization of YAP and TAZ and high expression of their target genes generally in breast cancers with a poor outcome [8–11]. In contrast, little is known about the role of YAP/TAZ in the initial stages of human breast cancer formation. In the normal human mammary gland, TAZ is expressed at higher levels in the basal cells (BCs) that form the outer layer of the gland as compared with the cells of the inner luminal layer, and forced expression of TAZ in the luminal cells induces them to exhibit basal features [12]. In cells of the immortalized, but non-tumorigenic, human mammary MCF10A cell line, forced overexpression of YAP increased acinus-generating activity in three-dimensional (3D) cultures, and this response was further enhanced by a gain of AP-1 [13, 14]. However, early transforming events that take place in normal human mammary cells may not be adequately modeled by in vitro assays of genetically modified MCF10A cells, given the large transcriptional and epigenomic differences between MCF10A cells and freshly isolated subsets of normal human mammary cells [15].

We recently developed a highly efficient and reproducible method for rapidly generating serially transplantable invasive ductal carcinomas from either purified BCs or a progenitor-enriched subset of luminal cells (referred to as luminal progenitors or LPs) obtained directly from normal human reduction mammoplasty tissue. This method involves transducing the cells with a lentiviral vector encoding the cDNA of an oncogenic form of *KRAS* (*KRAS*^*G12D*^) followed by their immediate subcutaneous transplantation into immunodeficient female mice [16]. Using this protocol, we found that the progeny of the *KRAS*^*G12D*^-transduced cells show enhanced growth compared with controls within 2 weeks in vivo regardless of whether the initial cells are BCs or LPs, and within 6 weeks, small but palpable tumors are present. Histological analyses revealed these tumors to be phenotypically heterogeneous, containing variable numbers of cells positive for estrogen receptor alpha (ERα), heregulin-2 (HER2), epidermal growth factor receptor (EGFR), Ki67, and cytokeratins 8/18. In addition, clonal tracking experiments demonstrated that they are highly polyclonal [16].

Deregulation of the KRAS pathway has been genetically implicated in more than 20% of human breast cancers [17, 18], although oncogenic mutations in *KRAS* itself have been identified in only about 4% of cases [19]. This relevance of perturbed KRAS activity in human breast cancer, plus the efficiency, rapidity, and reproducibility of obtaining invasive ductal carcinomas from *KRAS*^*G12D*^-transduced primary human mammary cells prompted us to use this latter genetic model to investigate the role of YAP activity in the initiation of human breast cancers. The results identify an unexpected dependence of the initial phase of *KRAS*^*G12D*^-induced human mammary tumorigenesis on YAP inactivation, associated with an induced secretion of amphiregulin (AREG) that favors YAP phosphorylation. The significance of these unique findings using primary human cells is underscored by our confirmation of the opposite ability of YAP activation alone to promote the display of more transformed features by MCF10A cells in vitro, and an increased metastatic activity in vivo by MDA-MB-231 cells, a highly tumorigenic established human breast cancer cell line with a similar KRAS mutation.

## Results

### YAP expression is downregulated in *KRAS*^*G12D*^-transduced normal human mammary cells

**Fig. 1:**
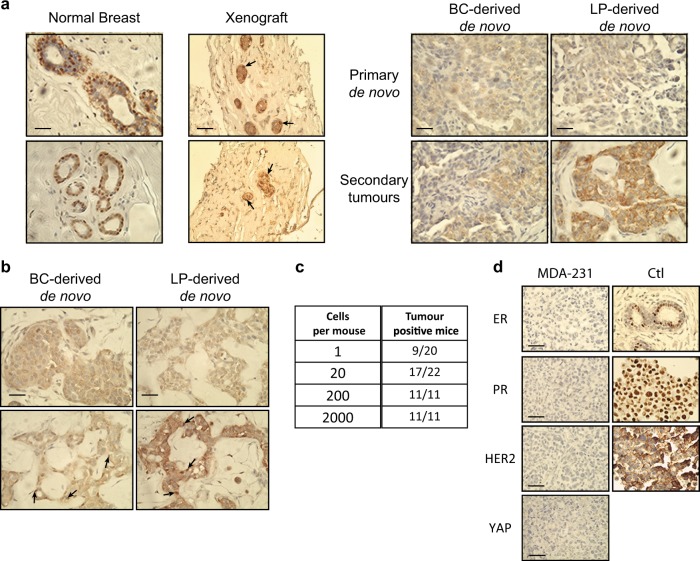
*KRAS*^*G12D*^-induced transformation of purified isolates of primary human mammary cells leads to YAP inactivation. **a** Representative views of immunostaining of YAP in normal human mammary tissue (two donors, left panel) or mammary structures produced from normal human mammary cells in collagen gels transplanted under the kidney capsule of NRG mice (two donors, middle panel), or 8-week primary and secondary *KRAS*^*G12D*^-derived tumors initiated from BCs or LPs injected subcutaneously with Matrigel into female NRG mice (right); *N* = 4 donors. Scale bar, 100 μm. **b** Representative views of YAP immunostaining of cells recovered from NRG mice injected subcutaneously with Matrigel 2 weeks previously with *KRAS*^*G12D*^-transduced human mammary cells; *N* = 3 donors. Scale bar, 100 μm. **c** Table showing total number of cells used to initiate each tumor, and the frequency of tumor positive mice. **d** Representative views of immunostained ERα, PR, HER2, and YAP in tumors-derived from MDA-MB-231 cells (top panel). Control sections are from normal human breast tissue (ERα), T47D cells (PR), and SKBr3 (HER2) cell lines. Scale bar, 100 μm

In a first series of studies, we used immunohistochemistry (IHC) to compare the expression of YAP in sections of normal breast mammoplasty reduction tissue and in primary tumors produced from *KRAS*^*G12D*^-transduced normal human mammary cells transplanted into female nonobese diabetic-*Rag1*^−/−^-*IL2Rγc*^−/−^ (NRG) mice. These analyses included tumors derived from two phenotypically separable and biologically distinct subpopulations of cells in the human mammary gland: EpCAM^low/−^CD49f^+^ BCs and EpCAM^+^CD49f^+^ LPs, purified from freshly dissociated reduction mammoplasty tissue and transduced as previously described [16]. The results for normal human breast tissue showed YAP to be largely restricted to the nuclei of cells in the basal layer (Fig. 1a, left panel), as expected from the previously reported expression of TAZ [12, 20] and an oppositely restricted expression of the YAP regulator LATS1/2 in cells of the luminal layer [20].

In marked contrast, YAP was either undetectable or cytoplasmic in all examined 8-week tumors generated from the *KRAS*^*G12D*^-transduced primary human mammary cells, both BCs and LPs (Fig. 1a, right panel, and Supplementary Fig. 1). A loss of nuclear YAP was also seen in secondary passages of similarly derived tumors (Fig. 1a and Supplementary Fig. 1). IHC examination of lesions present in mice 2 weeks after they had been transplanted with *KRAS*^*G12D*^-transduced cells revealed an already altered phenotype with prevalent cytoplasmic YAP and only occasional cells with detectable nuclear YAP (Fig. 1b).

To control for potential effects of analyzing cells that had been obtained from enzymatically dissociated mammary tissue and then stimulated to grow in transplanted mice, we performed similar IHC staining of the normal structures that form within 4 weeks from non-transduced, but fully dissociated normal human mammary cells (together with irradiated mouse fibroblasts) in collagen gels transplanted into NRG mice [21, 22]. These analyses showed a predominant expression of YAP in the nucleus of the regenerated BCs, indicating the lack of YAP seen in the *KRAS*^*G12D*^-induced transformants is not simply a consequence of a stimulated regenerative process (Fig. 1a).

To compare this unexpected change in YAP expression with another model of human breast cancer with *KRAS* mutations, we first examined tumors similarly generated in female NRG mice 8 weeks after being injected with *KRAS*^*G12D*^-transduced MCF10A cells [23]. IHC of these tumors also showed a lack of YAP (Supplementary Fig. 1). For a more advanced model, we examined tumors produced from MDA-MB-231 cells (with a *KRAS*^*G13D*^ mutation [24]) in female NRG host mice. Initial limiting dilution experiments showed that these cells have a very high content of tumor-initiating cells (one in nine cells using a 6-week endpoint, 95% CI = 1/6–1/14, Fig. 1c; and >1 in 3 cells with more prolonged follow up that showed 12 of 30 mice injected with single cells had developed palpable tumors within 13 weeks). IHC analysis of tumors produced from these cells also showed an absence of YAP in addition to ER, PR, and HER2 negativity (Fig. 1d).

Taken together, these results demonstrate an early and subsequently sustained suppression of YAP expression in tumors generated in vivo by multiple models of transformed human mammary cells expressing an oncogenic form of KRAS.

### Initial *KRAS*^*G12D*^-induced tumorigenesis requires YAP downregulation, but subsequent metastatic activity is promoted by YAP activation

**Fig. 2:**
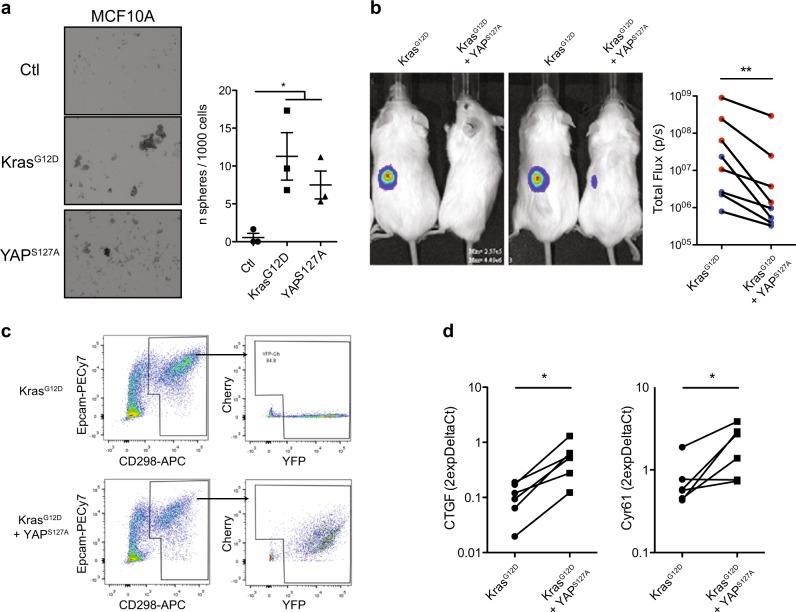
YAP inactivation is required for initial tumor formation by *KRAS*^*G12D*^-transduced normal human mammary cells. **a** Representative photomicrographs of MCF10A cells transduced with a *mCherry* only, or *KRAS*^*G12D*^-, or *YAP*^*S127A*^-*mCherry* vector and then cultured under mammosphere assay conditions for 7 days. Plot shows number of spheres generated from 1000 cells/cm^2^. *N* = 3. *P*-values are from Student’s *t*-test. **b** Representative photos of bioluminescence signals of NRG mice injected subcutaneously 2 weeks previously with Matrigel and *KRAS*^*G12D*^-transduced human mammary cells or human mammary cells co-transduced with both *KRAS*^*G12D*^ and *YAP*^*S127A*^ vectors. Plot shows the bioluminescence activity from donor- and cell type-matched pairs of primary tumors (from BCs in blue; from LPs in red); *N* = 4 donors. *P*-values are from a paired *t*-test comparing matched starting cells transduced with *KRAS*^*G12D*^ with or without *YAP*^*S127A*^. **c** Tumors generated from cells transduced with *KRAS*^*G12D*^ only, or *KRAS*^*G12D*^ + *YAP*^*S127A*^ were dissociated and analyzed for their content of human (CD298/EpCAM)^+^ and Cherry (YAP^S127A^)^+^ and/or YFP (KRAS^G12D^)^+^ cells. **d** *CTGF* and *CYR61* mRNA levels in tumors generated from *KRAS*^*G12D*^ and *KRAS*^*G12D*^ + *YAP*^*S127A*^-transduced cells. *GAPDH* mRNA was used to normalize the RNA content of each sample; *N* = 4 donors. *P*-values are from Student’s paired *t*-test comparing matched starting cells transduced with *KRAS*^*G12D*^ with or without *YAP*^*S127A*^

To determine if and how the prevalent absence of YAP (and hence its inferred transcriptional control properties) in human mammary cells with oncogenic *KRAS* mutations may affect the transformed properties they display, we examined their behavior when forced to express a constitutively active form of YAP. Accordingly, we constructed a lentiviral vector encoding a *YAP*^*S127A*^ cDNA which is not subject to LATS kinase-mediated phosphorylation and hence is retained in the nucleus where it continuously mimics the transcriptional activation properties of wild-type YAP [25]. Transduction of MCF10A cells with WT-*YAP* was previously reported to enhance their growth or ability to generate spheres in vitro [13, 14]. Using this assay, we confirmed the same effect was obtained on MCF10A cells transduced with our *YAP*^*S127A*^ vector (Fig. 2a), even though the tumor spheres were smaller than those obtained from MCF10A cells transduced with *KRAS*^*G12D*^.

We then transduced purified BCs and LPs with either one or both the *KRAS*^*G12D*^ and *YAP*^*S127A*^ vectors as well as a luciferase vector, and then transplanted each different group of cells into separate groups of female NRG mice (~1000 cells/mouse). Bioluminescence monitoring of these mice showed a consistently decreased signal from the *KRAS*^*G12D*^ and *YAP*^*S127A*^ co-transduced transplants as compared with transplants of cells from the same subset of cells from the same donor and transduced with *KRAS*^*G12D*^ alone (Fig. 2b). The tumors generated from the co-transduced cells also showed the evidence of greater YAP activity (higher content of transcripts of *CTGF* and *CYR61*, two well-known YAP/TAZ target genes), in addition to being smaller in size by comparison with tumors produced from the same cells transduced with *KRAS*^*G12D*^ alone (Fig. 2c, d). *YAP*^*S127A*^ alone did not confer tumorigenic activity on either BCs or LPs (Supplementary Fig. 2).

**Fig. 3:**
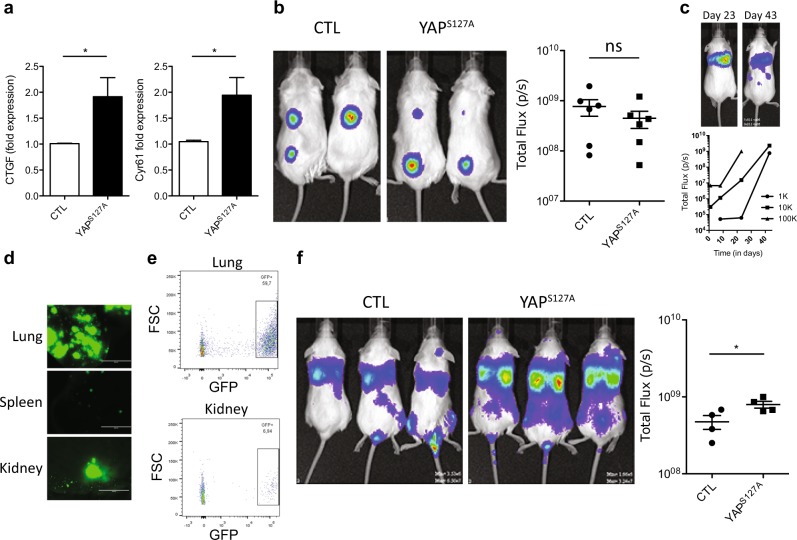
Forced YAP activation promotes the formation of metastases from IV-injected MDA-MB-231 cells. **a** *CTGF* and *CYR61* mRNA levels from control- or *YAP*^*S127A*^-transduced MDA-MB-231 cells. *GAPDH* mRNA was used to normalize for the RNA content in each sample. **b** Representative pictures of bioluminescence signals from NRG mice injected subcutaneously with Matrigel and 1000 MDA-MB-231 cells transduced with empty vector or *YAP*^*S127A*^, and a plot showing the bioluminescence results. **c** Representative pictures of bioluminescence signals in NRG mice injected IV with 1000 MDA-MB-231 cells 23 and 43 days previously (Top panel). Graph showing bioluminescence signals from mice injected IV with 1, 10, or 100 K MDA-MB-231 cells (bottom panel). **d** Representative pictures (EVOS imaging system) of the lung, spleen and kidney, from mice injected IV with 1000 MDA-MB-231 cells. **e** GFP^+^ cell content of cell suspensions prepared from dissociated lungs and kidneys obtained from mice described in **d** and analyzed by flow cytometry. **f** Shown are representative pictures of bioluminescence signals from NRG mice injected IV with MDA-MB-231 transduced with empty vector or *YAP*^*S127A*^, and a plot showing the bioluminescence results

We also examined the effect of forced expression of *YAP*^*S127A*^ in MDA-MB-231 cells. Molecular analysis showed the expected increased expression of *CTGF* and *CYR61* in the transduced cells (Fig. 3a), but standard transplants in female NRG mice showed no evidence of an effect on the rate of growth of tumors generated from them at the site of injection (Fig. 3b). To query potential effects on the known ability of MDA-MB-231 to generate metastases in the lung, spleen, and kidney in intravenously (IV) injected mice, we also assessed the effect of forced *YAP*^*S127A*^ expression in MDA-MB-231 cells using this route of transplantation. The results showed an increased metastatic ability of the *YAP*^*S127A*^-transduced MDA-MB-231 cells compared with control-transduced cells (Fig. 3c–f).

Taken together, these findings reveal that the effect of YAP on the tumorigenic properties of *KRAS*^*G12D*^-transduced human mammary cells is highly context-dependent with a strong dependence on YAP inactivation exclusive to the initial phase of transformation, and an opposite ability of increased YAP activity to promote the metastasis of cells with a more advanced malignant phenotype.

### KRAS^G12D^ promotes YAP inactivation in primary human mammary cells by inducing secretion of AREG

**Fig. 4:**
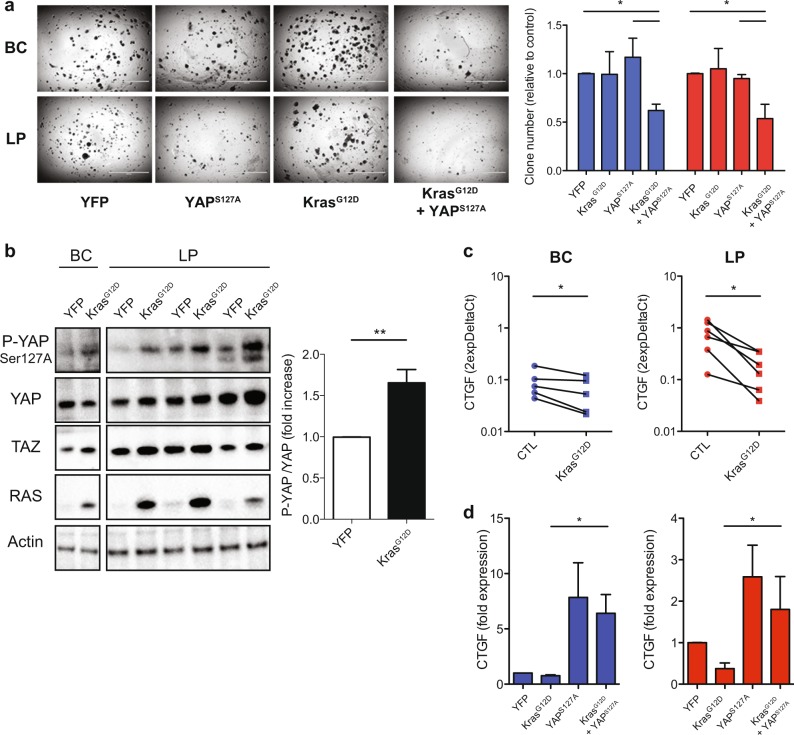
KRAS transformation of primary human mammary cells leads to YAP inactivation in vitro. **a** Representative pictures from 14-day 3D cultures of human BCs and LPs transduced with *YFP*, *YAP*^*S127A*^, *KRAS*^*G12D*^, or *KRAS*^*G12D*^ + *YAP*^*S127A*^. Bar graphs show clone numbers (relative to YFP control); *N* = 3 donors. **b** Western blots showing YAP phosphorylation (Ser127) levels (relative to YAP) in control and *KRAS*^*G12D*^-transduced human BCs and LP cells assessed 3 days post transduction; bar graph showing P-YAP/YAP ratio (Actin used as internal control) in human BCs and LPs transduced with *YFP*, or *KRAS*^*G12D*^; *N* = 3 donors. **c** *CTGF* and *CYR61* mRNA levels from control and *KRAS*^*G12D*^-transduced human BCs and LPs assessed 3 days post transduction. *GAPDH* mRNA was used to normalize the RNA content in each sample; *N* = 5 or 6 donors. *P*-values are from a paired *t*-test comparing matched starting cells transduced with *KRAS*^*G12D*^ with or without *YAP*^*S127A*^. **d** *CTGF* mRNA levels from BCs (left, blue) and LPs (right, red) transduced with *YFP*, *KRAS*^*G12D*^, *YAP*^*S127A*^, or *KRAS*^*G12D*^ + *YAP*^*S127A*^. *GAPDH* mRNA was used as a normalizer for each sample. *N* = 3 donors

We next sought to investigate the mechanism by which *KRAS*^*G12D*^ causes a loss of YAP activity in primary human mammary cells. Examination of the immediate effects of transducing isolated BCs and LPs with *YAP*^*S127A*^ as well as *KRAS*^*G12D*^ (Supplementary Fig. 3a) showed that this manipulation caused a significant decrease in the in vitro clonogenic activity of these cells in 2D (Supplementary Fig. 3b) as well as 3D (Fig. 4a) assays. *KRAS*^*G12D*^ alone caused an increase in the level of phosphorylated YAP (p-YAP) in both cell types evident within 3 days (Fig. 4b), and an accompanying decrease in *CTGF* transcripts (Fig. 4c). In contrast, primary cells transduced with *YAP*^*S127A*^ as well as *KRAS*^*G12D*^ did not show a decrease in *CTGF* mRNA (Fig. 4d). Together, these findings demonstrate that *KRAS*^*G12D*^-induced phosphorylation of YAP is an important component of the mechanism responsible for the loss of YAP activity in *KRAS*^*G12D*^-transduced BCs and LPs and the enhanced proliferative activity of their progeny.

**Fig. 5:**
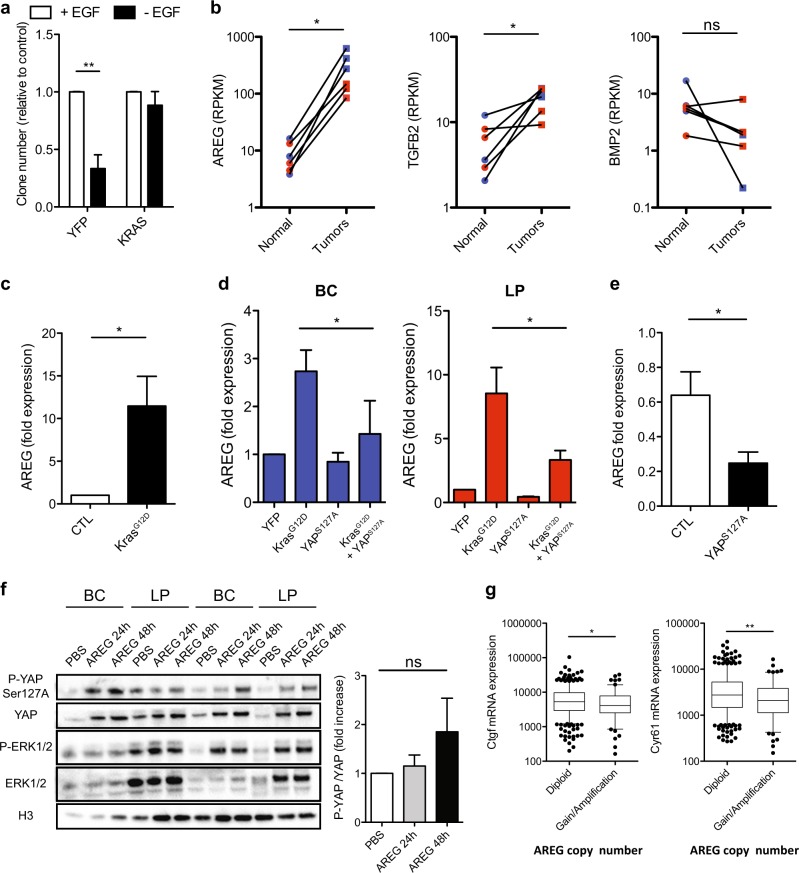
*KRAS*^*G12D*^ promotes *AREG* expression and inactivation of YAP. **a** Bar graphs showing total cell numbers in cultures of control and *KRAS*^*G12D*^-transduced human BCs and LPs assessed 4 days post transduction, with or without 20 ng/ml of EGF (values are shown relative to the +EGF condition); *N* = 3 donors. **b** RNAseq data from normal human subsets versus de novo tumors (BCs and BC-derived tumors in blue; LPs and LP-derived tumors in red). Values for *AREG*, *TGFβ2*, and *BMP2* are shown as RPKMs; *N* = 3 donors. *P*-values are from paired *t*-tests for each comparison. **c** *AREG* mRNA levels from control and *KRAS*^*G12D*^-transduced human BCs and LPs assessed 3 days post transduction. *GAPDH* mRNA was used to normalize the RNA content in each sample; *N* = 6 donors. **d** *AREG* mRNA levels from human BCs (left, blue) and LPs (right, red) transduced with *YFP*, *KRAS*^*G12D*^, *YAP*^*S127A*^, or *KRAS*^*G12D*^ + *YAP*^*S127A*^; *N* = 3 donors. **e** *AREG* mRNA levels of MDA-MB-231 ± *YAP*^*S127A*^. *GAPDH* mRNA was used to normalize the RNA content in each sample. *N* = 3. **f** Western blot comparing YAP phosphorylation (Ser127) levels (relative to YAP) in human BCs and LPs assessed after 3 days in vitro with or without the addition of 50 ng/ml AREG (R&D systems) for 24–48 h; *N* = 3 donors. Bar graph showing P-YAP/YAP ratio (H3 used as internal control). **g** *CTGF* (left) and *Cyr61* (right) mRNA levels compared with *AREG* copy number status in invasive breast carcinoma samples in the TCGA dataset. Values for *YBX1* are shown as RPKMs

Previous reports have indicated that YAP activity can be influenced by BMP/TGFβ activation of Smad proteins [26, 27] as well as via AREG produced in response to EGFR activation [13]. Assessment of the in vitro clonogenic activity of *KRAS*^*G12D*^-transduced BCs and LPs immediately post transduction indicated that their EGF dependence was already markedly reduced compared with control-transduced cells (Fig. 5a). To look for candidate indicators of the mechanism responsible, we surveyed the differentially expressed transcripts evident in previously published RNAseq data for normal human mammary BCs and LPs and their matched *KRAS*^*G12D*^-derived transformants [16]. These comparisons revealed a 10–40-fold increased expression of *AREG* transcripts in tumors derived from either BCs or LPs. *TGFβ* transcript isoforms were also upregulated in the tumor cells, but to a lesser extent, and *BMP* transcript levels were neither markedly nor consistently altered (Fig. 5b and Supplementary Fig. 4a). The quantitative reverse transcription-PCR (qRT-PCR) analysis of various transcripts in 3-day cultures of normal BCs and LPs that had been transduced with the *KRAS*^*G12D*^ (compared with a control vector) confirmed the levels of *AREG* transcripts to be selectively elevated in the *KRAS*^*G12D*^-transduced cells (Fig. 5c). In the same experiments, effects on *TGFβ2* and *BMP2* transcripts were inconsistent (Supplementary Fig. 4b). Interestingly, co-transduction of primary cells with both *YAP*^*S127A*^ and *KRAS*^*G12D*^ prevented the increase in AREG expression obtained with *KRAS*^*G12D*^ alone (Fig. 5d). In MDA-MB-231 cells, forced expression of *YAP*^*S127A*^ also decreased AREG expression (Fig. 5e), despite a lack of effect on their growth in vivo as tumors. Exposure of freshly isolated normal human mammary cells to AREG for 2 days in vitro also increased overall levels of p-YAP although the ratio of p-YAP/YAP was not altered (Fig. 5f). Finally, we examined the publicly available data for ~800 breast cancers in The Cancer Genome Atlas [28]. Interestingly, in this dataset, downregulated *CTGF* and *Cyr61* transcripts were associated with a gain of function or amplification of the *AREG* gene as compared with the patients whose tumors contained a normal diploid *AREG* complement (Fig. 5g).

Thus, forced expression of *KRAS*^*G12D*^ in normal human mammary cells rapidly increases p-YAP, and hence its inactivation, by a mechanism that involves the induced secretion of AREG.

## Discussion

Elucidating the perturbations of normal signaling pathways that contribute to the acquisition and expression of malignant properties by the cells that constitute human cancer is an area of growing importance. In this regard, the role of the YAP/TAZ complex that is part of the Hippo pathway and regulates many developmental processes [7] is gaining increasing attention. Notably, the expression of YAP/TAZ has recently been reported to be elevated in high-grade and triple-negative breast cancers by comparison with other subtypes, and is associated with their poor prognosis, tumor growth and metastatic ability [8–11]. TAZ also was found to promote the metastasis of aggressive patient-derived breast cancer cell lines [29] and, in pancreatic cancer, increased YAP could replace dependence on KRAS activity [30, 31]. Here we show that the forced expression of active YAP in MDA-MB-231 cells (a highly aggressive human breast cancer cell line that carries a mutant KRAS gene) also promoted their metastatic ability in transplanted immunodeficient mice.

However, little is known about the mechanisms that cause normal human mammary cells to initiate the process of tumorigenesis. An early study to address this question showed that the forced expression of a constitutively active or even wild-type form of YAP in non-tumorigenic but immortalized human MCF10A mammary cells enhanced their ability to grow in soft agar or mammosphere cultures [13, 14], that we confirmed here. These findings suggested that increased YAP activity might also play an important initiating role. However, our recent transcriptomic and epigenomic characterization of the three different mammary cell populations that constitute the normal adult human mammary gland and the stromal cells that surround it, as well as MCF10A cells, have shown MCF10A cells to be highly divergent from any of the freshly isolated human mammary cell types [15]. It was therefore of interest to re-examine initial changes that accompany the process of human mammary cell transformation in an experimental model in which tumors can be generated directly from primary cells. We have recently shown that this can be efficiently and reproducibly achieved by introducing *KRAS*^*G12D*^ into purified BCs or LPs isolated directly from normal human mammary glands by fluorescence activated cell sorting (FACS) [16]. We therefore chose this system to investigate the role of YAP in the early phase of mutant KRAS-mediated transformation of human mammary cells. The observation that inactivation of YAP, likely resulting from its KRAS-induced phosphorylation is critical to the process of KRAS-induced transformation of primary normal human mammary cells was therefore unexpected. However, despite the low level of YAP in unmanipulated MDA-MB-231 cells that also harbor a KRAS mutation, their opposite response to introduced YAP may be explained by their more complex mutational profile that include variants of *BRAF*, *CDKN2A*, and *TP53*, that could also interfere with YAP activity through alternative mechanisms [24, 32].

We also present evidence that this early phase of KRAS-induced transformation of human mammary cells involves activation of AREG production and the acquisition of EGF independence. It is therefore interesting that Britschgi et al. recently demonstrated that ERα levels in primary normal human cells are also regulated by LATS kinases and control mammary cell fate in part through both intrinsic and paracrine mechanisms via AREG secretion [20]. In accordance with these observations, we found that tumors generated de novo from *KRAS*^*G12D*^-transduced human mammary cells contain ERα^+^ cells and that *ESR1* expression is increased in both *KRAS*^*G12D*^-transduced BCs and LPs [16].

In summary, our findings serve to further illustrate the power and likely importance of de novo models of human tumorigenesis initiated with cells isolated directly from primary sources of normal human tissue. Such models can offer unique insights into mechanisms that may differentially affect early and late events in the process of tumorigenesis and that are not readily accessible to analysis from studies of cells from other species, patients’ samples or established human cancer cell lines [33].

## Materials and methods

### Cells and cultures

Normal reduction mammoplasty tissue was obtained with informed consent, using protocols approved by the University of British Columbia Research Ethics Board. The tissue was first dissociated to obtain organoid-rich pellets that were then viably cryopreserved [21]. Thawed organoids were rinsed with Hank’s Balanced Salt Solution supplemented with 2% fetal bovine serum (FBS) (HF), and the cells were then dissociated in 2.5 mg/ml trypsin with 1 mM EDTA and 5 mg/ml dispase (STEMCELL Technologies) with 100 μg/ml DNaseI (Sigma), with washing of the cells in HF between each step. The resulting cell suspension was filtered through a 40 μm mesh and BCs were isolated by FACS according to their CD45^−^CD31^−^EpCAM^lo^CD49f^+^ phenotype, and LPs according to their CD45^−^CD31^−^EpCAM^hi^CD49f^+^ phenotype as described [21]. Primary cells were cultured in SF7 media supplemented with 5% FBS. 2D CFC and 3D assays of human mammary cells were performed by culturing the cells in the presence of irradiated mouse 3T3 fibroblasts for 8, 10, or 14 days in SF7 media supplemented with 5% FBS as previously described [34].

MCF10A cells were obtained from J. Brugge (Harvard University, Cambridge, MA) and maintained in a 1:1 DMEM/F12 mix supplemented with 5% horse serum, 10 mg/ml insulin, 0.5 mg/ml hydrocortisone, 100 ng/ml cholera toxin, 20 ng/ml EGF (all Sigma), and 1% penicillin/streptomycin (Life Technologies). For mammosphere formation assay, 1000 cells/cm^2^ were seeded on ultra-low attachment plates (Costar), and mammospheres were counted after 1 week. MDA-MB-231 cells were obtained from S. Dunn (Child and Family Research Institute, Vancouver, BC) and maintained in DMEM with 10% FBS. Their identity was confirmed by DNA sequencing, including the detection of the *KRAS*^*G13D*^ allele [24]. T47D and SKBr3 cells were obtained from J. Emerman (University of British Columbia, Vancouver, BC) and maintained in DMEM with 10% FBS.

### Transduction and transfection

Primary cells were transduced with lentiviral vectors prepared and used as previously described [16]. In brief, primary BC and LP were transduced with lentiviral vectors coding for *YFP*, *YAP*^*S127A*^*-Cherry*, *KRAS*^*G12D*^-YFP or Luciferase, where *YAP*^*S127A*^ or *KRAS*^*G12D*^ were under MNDU3 promoter expression, and Luciferase, YFP or Cherry under PGK promoter expression.

### Xenografts

Female NRG mice were bred and housed in the specific pathogen-free animal facility in the British Columbia Cancer Research Centre. Surgeries were performed on 5–10-week-old mice. All procedures for the breeding and experimental use of mice were carried out using protocols approved by the University of British Columbia Animal Care Committee. To generate primary tumors, enzymatically dissociated suspensions of human mammary cells were prepared, purified by FACS, transduced and transplanted subcutaneously with 50% (v/v) Matrigel into mice [16]. To measure tumor bioluminescence from luciferase-expressing cells, mice were injected intraperitoneally with 150 mg/kg body weight of d-luciferin (Promega) and 10 min later the mice were imaged using a Xenogen IVIS Lumina system with Living Image version 3.0 software (Caliper Life Sciences). To prepare cell suspensions from tumors, the removed tissue was minced with a scalpel, incubated at 37 °C in DMEM/F12 media supplemented with 5% FBS, 300 U/ml collagenase, and 100 U/ml hyaluronidase (STEMCELL Technologies) for 1–2 h with periodic vortexing. The cells were then washed with HF, and treated with 2.5 mg/ml trypsin containing 1 mM EDTA, 5 mg/ml dispase, and 100 μg/ml DNaseI. Human cells were isolated by FACS after staining with anti-human-specific antibodies directed against human EpCAM and human CD298 (Biolegend) with simultaneous depletion of mouse cells stained with anti-mouse-specific antibodies directed against CD45 and CD31 (STEMCELL Technologies).

Subrenal xenotransplants were performed as previously described [21]. Briefly, human mammary BCs were combined with 10^5^ irradiated (50 Gy) C3H 10T1/2 fibroblast cells in a 25 μl volume of cold pH-neutralized rat tail collagen and placed into the individual wells of a 24-well plate. After the collagen gels had stiffened during a 10-min incubation at 37 °C, warm SF7 medium plus 5% FBS was added to the wells followed by incubation for another 50 min. The plates were then transferred to ice and the gels inserted under the kidney capsule through a 2- to 4-mm incision. An in house prepared slow-release pellet containing 2 mg of β-estradiol and 4 mg of progesterone (both from Sigma) was inserted subcutaneously in a posterior position. Four weeks after transplantation, mice were euthanized and the gels were removed aseptically from the kidneys and dissociated as described above for normal human mammary samples.

### Immunohistochemical staining

Pieces of normal breast tissue or tumors obtained from mice were fixed in 10% buffered formalin (Fisher), washed in 70% ethanol and embedded in paraffin. Sections of paraffin-embedded tissue (3 mm) were first treated with Target Retrieval solution (DAKO) and then a cytomation serum-free protein block (DAKO) followed by staining with human-specific antibodies recognizing YAP1 (1:200; Novus Biologicals, NB110-58358), ER (SP1; 1/50; Thermofisher; RM9101), PR (SP2; 1/50; Neomarker; 9102), and HER2 (4B5; Ventane). A secondary mouse antibody conjugated to horseradish peroxidase and treatment with 3,3ʹ-diaminobenzidine (DAB, DAKO) was used to obtain a positive brown staining. Negative IgG controls were performed on normal reduction mammoplasty tissue, and MDA-MB-231 cells served as positive control cells.

### Western blot and densitometry analysis

Treated cells were washed with cold PBS and incubated for 15 min at 4 °C with RIPA lysis buffer (30 mM Tris-HCl, pH 7.5, 150 mM NaCl, 10% glycerol, 1% Triton X-100 (Sigma) supplemented with a 1 mM NaF, 1 mM NaVO3, and 1 mM PMSF (all Sigma). Cell extracts were centrifuged at 13,000 × *g* for 10 min at 4 °C. The protein concentration of the supernatant fraction was then determined using the Bio-Rad Bradford Protein Assay Kit according to the manufacturer’s instructions. For each sample, an equal amount of total protein was diluted in sample buffer (Invitrogen) and boiled for 5 min. Samples were loaded onto precast 4–12% NuPAGE gradient polyacrylamide gels (Invitrogen). After electrophoresis, the proteins were transferred to a PVDF transfer membrane. Membranes were then blotted overnight at 4 °C with the appropriate primary antibodies, i.e., anti-Actin (Santa Cruz, sc-1615, 1/10000), anti-H3 (Cell Signaling Technology, 12648, 1/10000), anti-Ras (Cell Signaling Technologies, 3339, 1/1000), anti-TAZ (Cell Signaling Technology, 4583, 1/1000), anti-YAP1 (Novus Biologicals, NB110-58358, 1/1000), and anti-YAP^ser127^ (Cell Signaling Technology, 13008, 1/1000). Specific binding of antibodies was detected using appropriate secondary antibodies conjugated to horseradish peroxidase, and visualized with SuperSignal™ West Femto Maximum Sensitivity Substrate (Thermofisher), on ChemiDoc Gel Imaging system (Bio-rad). Densitometric analyses of immunoblots were performed using ImageJ.

### RNAseq data

RNAseq data were derived from Nguyen et al. [16] and expressed as reads per kilobase per million mapped reads (RPKM values). *P*-values were calculated using Student’s paired *t*-test.

### QRT-PCR

Total RNA was extracted from cryopreserved tumor samples or cultured cells using the Total RNA isolation Micro Kit (Agilent). cDNA was then synthesized using the SuperScript VILO cDNA synthesis kit (Life Technologies). QRT-PCR was performed using a SYBR Green master mix (Applied Biosystems) and samples run in triplicate with custom-designed primers (Table S1).

### Statistical analyses

Values are expressed as mean ± SEM, unless otherwise specified. Significance was evaluated using the Student’s *t*-test or paired *t*-test, as indicated, unless otherwise specified. *P < 0.05, **P < 0.01, ns = not significant.

## Supplementary information

Supplementary information and figures
