# Supplementary material for: Initiation of human mammary cell tumorigenesis by mutant KRAS requires YAP inactivation
Source: Oncogene. 2019 Nov 26;39(9):1957–68. doi: 10.1038/s41388-019-1111-0 (PMC7044112; doi:10.1038/s41388-019-1111-0)

Initiation of human mammary cell tumorigenesis by mutant KRAS requires YAP inactivation

Sylvain Lefort[1,*], Susanna Tan[1], Sneha Balani[1], Bo Rafn[2], Davide Pellacani[1], Martin Hirst[3], Poul H. Sorensen[2], Connie J Eaves[1].

[1]Terry Fox Laboratory, British Columbia Cancer Agency, 675 West 10th Avenue, Vancouver, BC V5Z 1L3, Canada.

[2]Department of Molecular Oncology, British Columbia Cancer Agency, 675 West 10th Avenue Vancouver, BC V5Z 1L3, Canada.

[3]Canada's Michael Smith Genome Sciences Centre, British Columbia Cancer Agency, Vancouver, BC V5Z 1L3, Canada; Michael Smith Laboratories, Department of Microbiology and Immunology, University of British Columbia, Vancouver, BC V6T 1Z4, Canada.

*Correspondence: sylvain.lefort@lyon.unicancer.fr

Running title: YAP inactivation requirement for KRAS mammary transformation

Figure S1. Representative views of immunostained YAP in 8-week primary and secondary tumors produced from freshly isolated normal human mammary BCs (blue) or LPs (red), or MCF10A cells after their transduction with $KRAS^{G12D}$; N = 4 donors. Site of transplantation is indicated as either subrenal or subcutaneous (SubQ).

Figure S2. Plot of the level of bioluminescence measured in the progeny of primary human BCs (blue) or LPs (red) transduced with YAP^{S127A}; N = 6 donors.

Figure S3. (**A**) Western blot showing YAP and RAS contents of cells generated *in vitro* from BCs and LPs transduced with *YFP*, YAP^{S127A}, $KRAS^{G12D}$, or $KRAS^{G12D}$ + YAP^{S127A}. N = 3 donors. (**B**) Bar graph showing CFC frequencies in human BCs and LPs transduced with *YFP*, YAP^{S127A}, $KRAS^{G12D}$, or $KRAS^{G12D}$ + YAP^{S127A} (relative to the YFP control), N = 3 donors.

Figure S4. (**A**) RNAseq data from normal human mammary BCs (blue circles), LPs (red circles) and tumors derived from BCs (blue squares) or LPs (red squares). Values for *TGFβ1*, *TGFβ3* and *BMP4* are shown as RPKM values. P-values are from paired t-tests comparing donor-matched data. (**B**) *TGFβ1*, *TGFβ2*, *BMP2* and *BMP4* mRNA levels from control- and $KRAS^{G12D}$-transduced human BCs and LPs assessed 3 days post-transduction. *GAPDH* mRNA was used to normalize for the RNA content of each sample.

Supplementary Figure S1

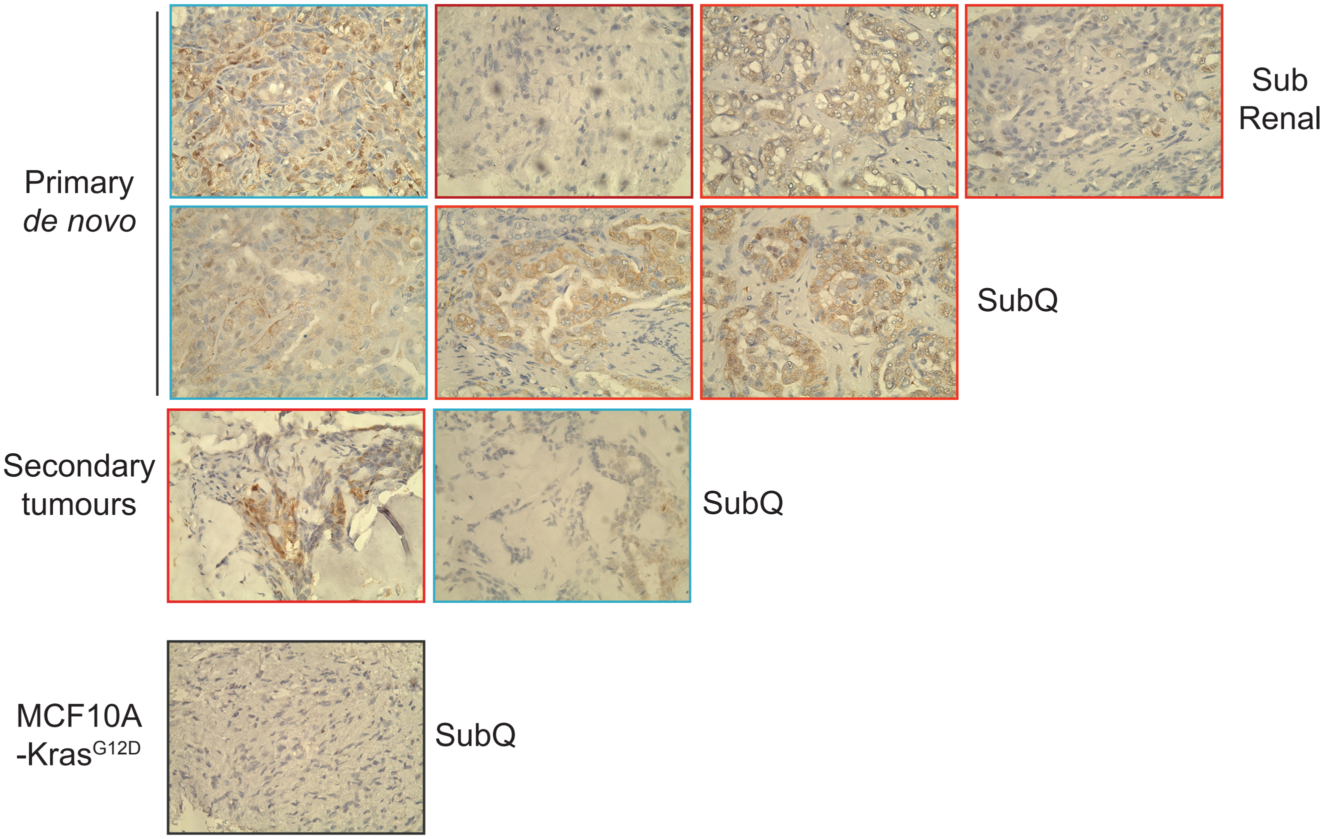

Supplementary Figure S2

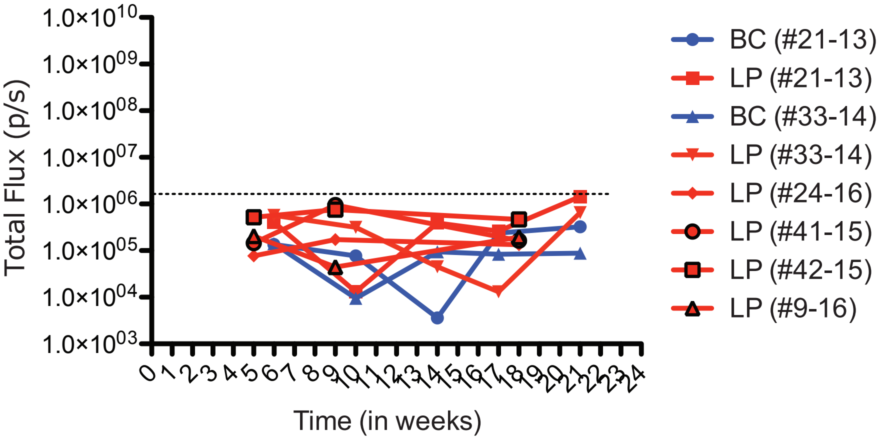

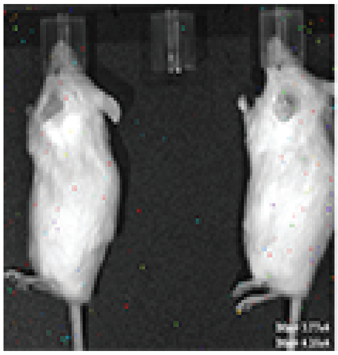

Supplementary Figure S3

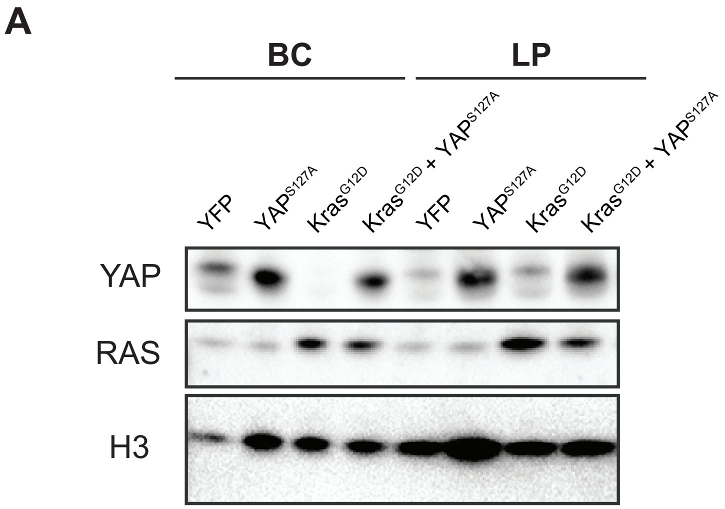

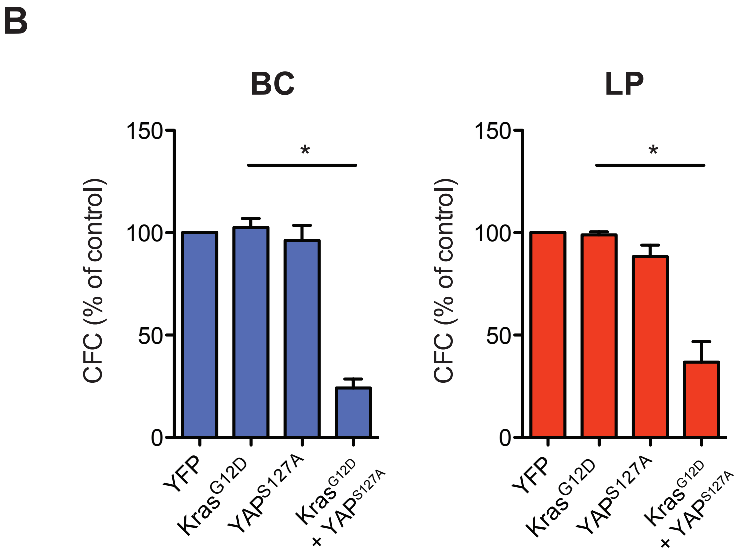

Supplementary Figure S4

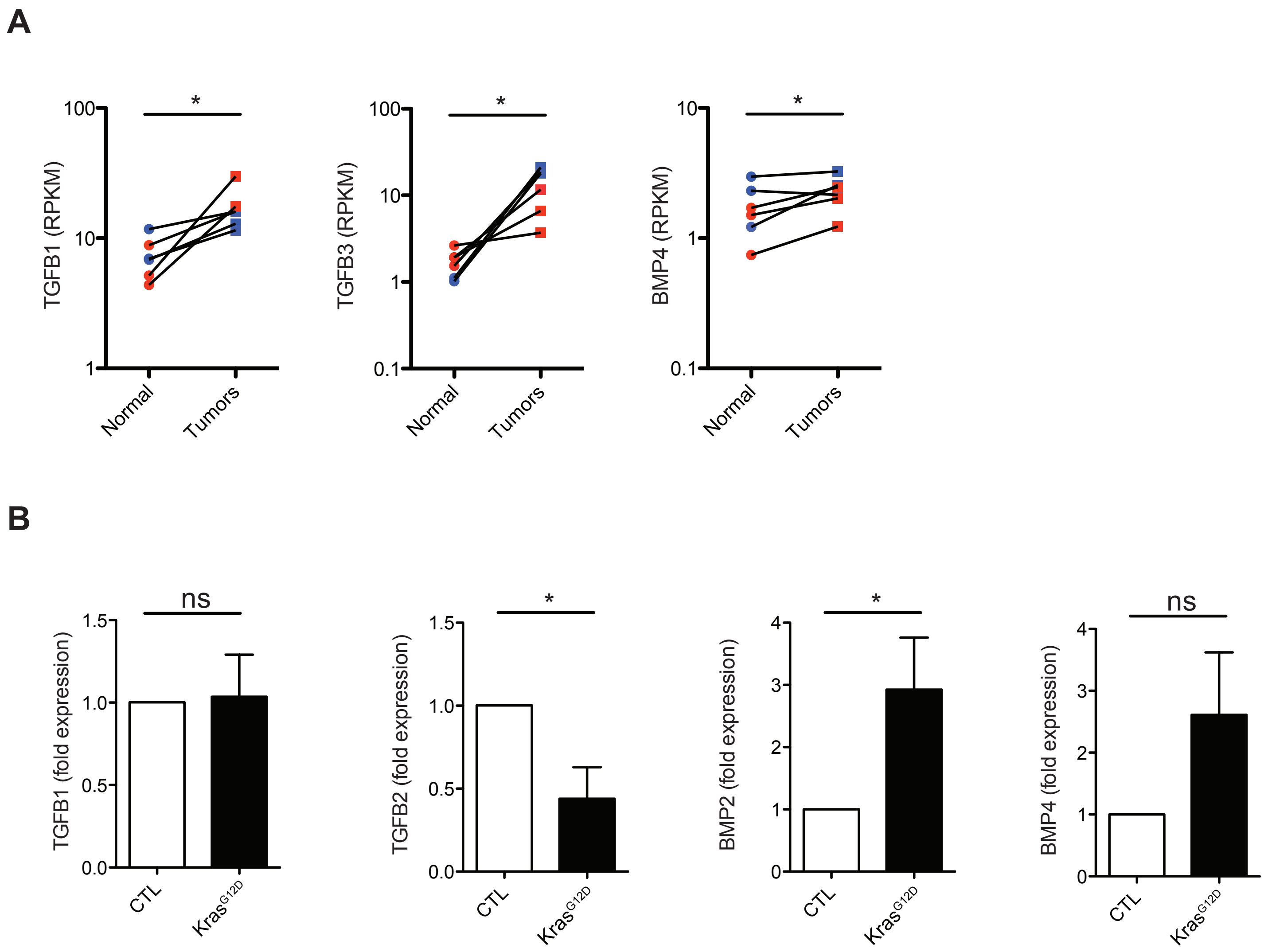

Supplement: Supplementary file 1 — Supplementary information and figures [file 41388_2019_1111_MOESM1_ESM.pdf]
